# Beyond the Badge: A Scoping Review Among Police Personnel in South India Exploring the Impact of Yoga and Health Education on Cardiovascular Health

**DOI:** 10.7759/cureus.68370

**Published:** 2024-09-01

**Authors:** Angusubalakshmi R, Pooja Mary Vaishali, Gomathy Parasuraman

**Affiliations:** 1 Department of Community Medicine, Saveetha Medical College and Hospital, Saveetha Institute of Medical and Technical Sciences, Saveetha University, Chennai, IND

**Keywords:** world health organization (who), public health care, cvd risk factors, police personnel, non communicable disease (ncd)

## Abstract

Communicable diseases have been the primary cause of morbidity and mortality, affecting populations for decades. However, in recent times, noncommunicable diseases (NCDs) have emerged as the primary cause of illness and premature death due to factors such as urbanization, longer life expectancy, and unhealthy lifestyles. In recent years, noncommunicable illnesses have emerged as the primary cause of morbidity and premature mortality, replacing infectious diseases as the leading cause of illness and death. Among the top five causes of NCD, cardiovascular disease (CVD) is the most important factor, comprising the major diseases with maximum mortality and morbidity. The burden of CVD is greatly increased by modifiable risk factors, such as smoking, high blood pressure, type 2 diabetes, low-density lipoprotein cholesterol, and excess body weight.

CVD occurs particularly in certain occupational risk groups, such as doctors, police personnel, and persons working for prolonged hours, predisposing them to unhealthy dietary practices, improper sleeping patterns, and increased psychological stress. As members of this occupational group, police personnel are particularly at risk for cardiovascular diseases, making it imperative to implement preventive measures to reduce the burden of these diseases in this population. The primary objective was to assess the impact of yoga and health education interventions on cardiovascular health outcomes among police personnel in South India, and the secondary objective was to examine the changes in blood pressure levels and lipid profiles following yoga and health education programs among police personnel.

## Introduction and background

Cardiovascular diseases (CVDs) are influenced by a combination of genetic, environmental, and lifestyle factors, with atherosclerosis being the central component. Atherosclerosis is a chronic inflammatory condition characterized by the accumulation of cholesterol-rich plaques in arterial walls, which can result in myocardial infarction, stroke, or other cardiovascular events. Several modifiable risk factors significantly contribute to the development of CVDs, including hypertension, dyslipidemia, diabetes mellitus, smoking, unhealthy diet, inadequate physical inactivity, and excessive alcohol consumption [1]. These risk factors promote atherosclerosis progression and increase the likelihood of adverse cardiovascular events. Despite advances in treatment, CVDs pose significant public health challenges and are a major cause of mortality and disability worldwide. Effective prevention and management of CVDs require addressing individual risk factors and broader social determinants of health. These include living and working conditions, access to healthcare, education, and income, all of which can affect CVD risk.

Additionally, first responders, including healthcare professionals, police officers, drivers, and other workers in high-stress, irregular-hour, and trauma-exposed jobs, are at significant risk for CVD because of factors such as sleep disturbance, unhealthy habits, and exposure to environmental hazards. Working in shifts can disrupt the body's natural circadian rhythms, leading to sleep problems and metabolic issues, in addition to stress, long hours, and physical demands, which can increase the risk of hypertension, obesity, and other factors that contribute to CVD [2]. Workers in various professions, including rotating or night shifts, office workers, construction workers, factory workers, miners, extraction workers, military personnel, and retail workers face similar risks owing to their sedentary lifestyles, prolonged sitting, reduced physical activity, exposure to environmental hazards, repetitive motion, standing and noise/chemical exposure. These factors can contribute to increased CVD risk in these workers [3]. To decrease the likelihood of CVD among workers in specific professions, it is crucial to implement targeted interventions, such as workplace wellness programs, education about CVD risk factors, and methods to promote healthy lifestyles and reduce occupational hazards.

Police officers face a higher risk of CVD because of their demanding and stressful work conditions, prolonged inactivity, intense activity, irregular hours, and exposure to traumatic events [4]. These factors lead to high levels of psychological stress, limited access to healthy lifestyle resources and preventive healthcare, and increased rates of hypertension, obesity, dyslipidemia, and metabolic syndrome. Unhealthy behaviors, such as smoking, an unbalanced diet, and insufficient physical activity, further exacerbate the CVD risk in police personnel, resulting in higher morbidity, premature disability, and reduced life expectancy. Improving population-wide preventive measures to address the root causes of CVDs is crucial. Encouraging healthy lifestyles, regular physical activity, balanced diets, smoking cessation, and moderate alcohol consumption are the key approaches for preventing CVDs [5]. To achieve this, comprehensive preventive and management approaches that target modifiable risk factors and enhance psychosocial well-being are necessary. Incorporating health education and yoga therapy into the workplace can help achieve these goals. The WHO Health Organization has identified NCDs as a growing concern, which has led the United Nations to set global targets for their prevention and management. In response, India's national policy has prioritized reducing premature deaths from CVD and controlling hypertension and diabetes [6,7]. To achieve these goals, early detection and management of CVD risk factors, maintenance of a balanced diet, and regular physical activity are crucial. Health education plays a vital role in empowering individuals with the knowledge to make informed health choices and adopt healthier behaviors. This includes raising awareness of cardiovascular risk factors and promoting lifestyle modification. Numerous randomized controlled trials (RCTs) and observational studies have demonstrated the efficacy of health education programs in reducing cardiovascular risk factors and improving outcomes. Such programs generally encompass dietary counseling, physical activity promotion, stress management, and smoking cessation support, resulting in significant reductions in blood pressure, cholesterol, body weight, and overall cardiovascular risk.

Yoga therapy is a valuable adjunct approach for managing cardiovascular CVD risk factors and improving cardiovascular health. This therapeutic approach incorporates techniques, such as meditation, mindfulness, and controlled breathing, to decrease stress levels, which are significant CVD risk factors. The National Programme for Prevention and Control of Diabetes, Cardiovascular Diseases, and Stroke (NPCDCS) in India aims to alleviate the NCD burden, particularly among productive-age groups. Furthermore, physical postures in yoga improve flexibility, strength, balance, and weight management, as well as circulation and cardiovascular fitness [8,9]. Some yoga practices also aid in regulating blood pressure, and regular practice can lower systolic and diastolic blood pressure, thereby reducing the risk of hypertension and CVD. The emphasis on relaxation and mindfulness in yoga therapy also promotes better sleep quality, which supports cardiovascular health. In addition, yoga therapy encourages dietary improvement, smoking cessation, and other lifestyle changes that promote heart health. It also enhances mental health by reducing the symptoms of depression and anxiety, and improving overall emotional well-being, all of which benefit cardiovascular health. However, it is crucial to use yoga therapy in conjunction with conventional medical treatments and to seek supervision from healthcare professionals. It is essential to find a suitable style and intensity level of yoga for individual needs and abilities, as not all forms of yoga are appropriate for everyone. 

## Review

Rationale of the study

This review aimed to explore the impact of yoga and health education on the cardiovascular health of police personnel in India, taking into account their heightened susceptibility to non-communicable diseases, especially cardiovascular diseases. By examining the potential of yoga as a holistic practice to reduce these risks and enhance overall health outcomes, this review aims to provide valuable insights.

Eligibility criteria

The research papers included in this study were chosen based on the following criteria: (i) research focused on cardiovascular disease risk among police personnel; (ii) publications written in English; (iii) papers discussing interventions, yoga practices, and health education; (iv) experimental and observational studies, including randomized controlled trials, cohort studies, and cross-sectional studies on CVD; and (v) full-text articles that were accessible for review. The final papers were selected based on the highest quality of research design (scoping and systematic review), publications by renowned international scholars, studies using qualitative research designs, and the date of publication. The findings were combined and presented under the heading of cardiovascular disease risk among police personnel and interventions, such as yoga and health education, to reduce the risk of cardiovascular disease.

Information sources

**Figure 1:**
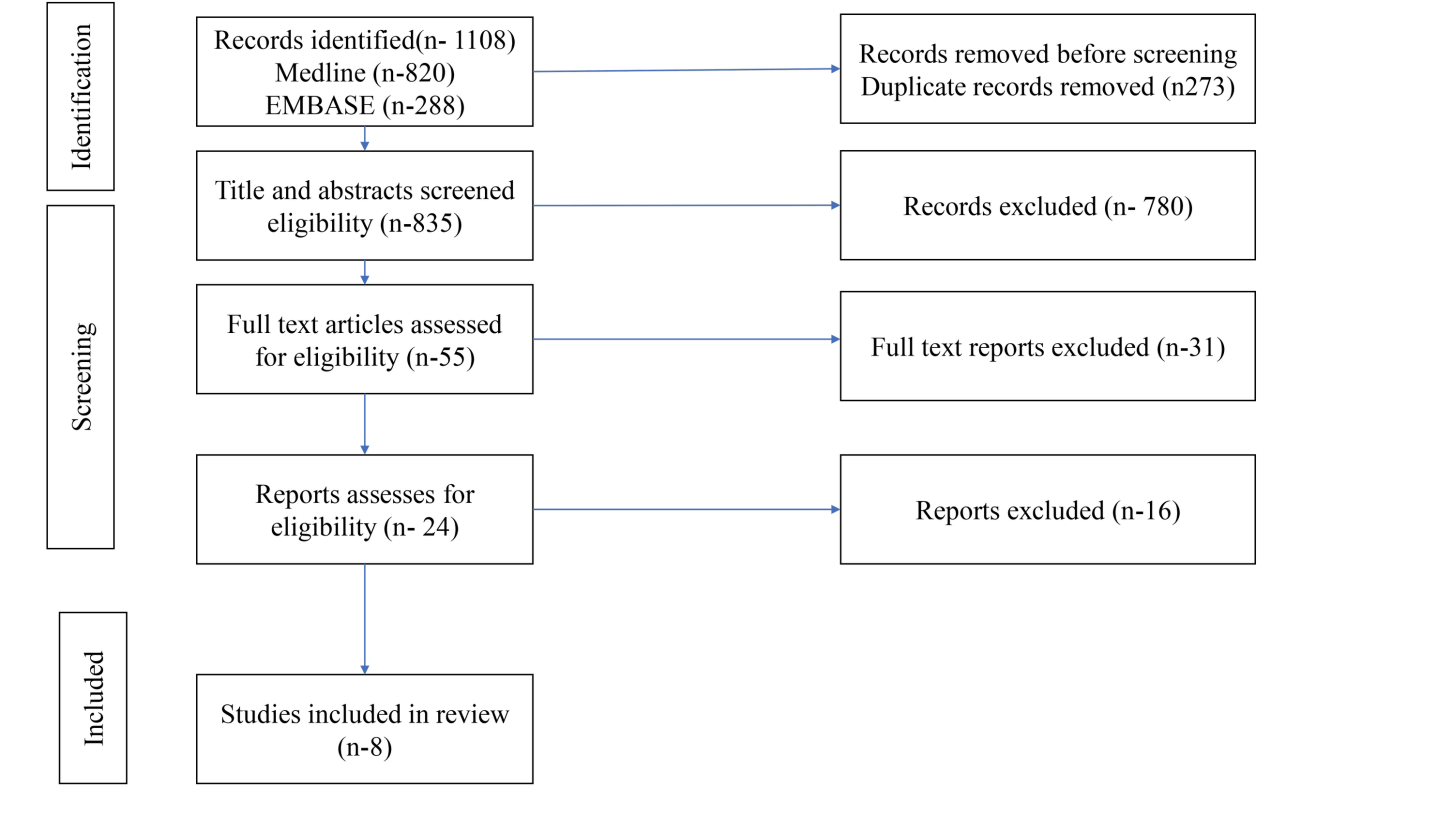
PRISMA flow chart PRISMA - Preferred Reporting Items for Systematic Reviews and Meta-Analysis

The initial search yielded 1108 citations, which were then screened using predetermined eligibility criteria. After the removal of 273 duplicates, 835 studies were screened. These studies were then screened using predetermined eligibility criteria, and 780 articles were excluded for the following reasons: 370 for being on the wrong topic, 172 for being on the wrong publication type (e.g., abstract only, dissertations), 89 for being too old, 52 for focusing on the wrong population, 58 for not aligning with the review's objectives and 31 for having the wrong study design. Ultimately, eight articles were included in the final review. The research papers included in this study were chosen based on the following criteria: research focused on cardiovascular disease risk among police personnel, publications written in English, papers discussing interventions, yoga practices, and health education; experimental and observational studies, including randomized controlled trials, cohort studies, and cross-sectional studies on CVD; full-text articles that were accessible for review; and the final papers were selected based on the highest quality of research design (scoping and systematic review), publications by renowned international scholars, studies using qualitative research designs, and the date of publication. The findings were synthesized and presented under the heading of cardiovascular disease risk among police personnel and interventions, such as yoga and health education, aimed at reducing the risk of cardiovascular disease. The screening and selection processes are illustrated in Figure 1.

Selection strategy

The Medical Subject Headings (MeSH) phrases found in the PubMed databases, such as MEDLINE, were utilized to gather pertinent information about the risk of cardiovascular disease in law enforcement officers, as well as the strategies already in use to prevent it from developing. The search was limited to articles published between 2015 and 2023, and the following MeSH terms were utilized: "cardiovascular disease risk", "high-risk groups for CVD", "universal health coverage", "police personnel", "yoga therapy and CVD", "health education and CVD", "non-communicable diseases", and "India". The findings were combined and presented under the heading of cardiovascular disease risk among police personnel and interventions, such as yoga and health education, to reduce the risk of cardiovascular disease.

Epidemiological transition

The term "epidemiological transition" describes the shift that takes place in developing nations when mortality rates from infectious diseases and malnutrition fall and those from non-communicable diseases rise. In nations where the risk factors resulting from dietary, environmental, and physical activity changes have increased, this shift in disease and death rates is related to economic expansion, urbanization, industrialization, and changes in social structures [10]. Globalization of eating patterns, characterized by the rising consumption of fats and sweets, as well as urbanization, has contributed to the overall epidemiological shift. According to assessments, India's prevalence of coronary heart disease has risen dramatically over the last three decades, from 1.6% to 7.4% in rural areas and from 1% to 13.2% in urban areas. Sustainable Development Goals place a strong emphasis on the necessity of multi-sectoral national plans aimed at preventing and reducing the burden of non-communicable diseases (NCDs). India's 2017 national strategy for the integrated management of NCDs aims to identify and treat 80% of patients with diabetes and hypertension and to reduce premature deaths from CVDs by 25% [11]. Non-communicable diseases (NCDs) account for 53% of fatalities and 44% of disability-adjusted life-years lost in the United States, making them a major cause of mortality and life-years lost due to disability. Among the various occupational risk groups, police officers are undeniably one of the most vulnerable demographics for NCDs, placing them in the high-risk category. This can be attributed to factors such as long working hours and stressors, which contribute to high levels of stress and pressure. Furthermore, police officers often have limited food options and restricted diets while on duty, leading to higher rates of stress, disrupted sleep patterns, and increased alcohol and tobacco use. Research has indicated that there is a greater incidence of type 2 diabetes and cardiovascular disease risk factors in this occupational category [12].

CVD and police personnel

A study conducted on US police personnel found that they are more likely to develop non-communicable diseases and experience cardiovascular events at a younger age than other occupational groups. This group also had a higher rate of premature death than the other groups. In India, the police force is divided into constables, inspectors, and officers, with constables being the lowest social stratum who perform tasks assigned to them as part of police activity and follow orders from inspectors and sub-inspectors. Physical fitness is essential for all public safety workers, including police officers, because they may face demanding physical demands while on active patrols. The National Program for Prevention and Control of Non-communicable Diseases (NPNCD), which was established by the Ministry of Health and Family Welfare in response to the high prevalence of non-communicable diseases in India, includes yoga as a crucial part of its interventions to combat non-communicable diseases. Non-communicable diseases disproportionately affect productive age groups, which is a cause for concern. Given the increased risk of cardiovascular diseases, it is crucial to implement preventive measures to reduce the burden of these diseases among police personnel [13].

Interventions to reduce CVD

Interventions for cardiovascular disease can involve altering lifestyle habits such as consuming a nutritious diet, participating in regular physical activity, refraining from smoking, managing stress levels, and maintaining a healthy body weight. Furthermore, medical interventions may involve administering medications to regulate blood pressure, cholesterol levels, and blood sugar, as well as implementing procedures such as angioplasty or bypass surgery in more severe cases. The effective management of other risk factors, including diabetes and hypertension, is essential. The results of this investigation highlight the significance of taking preventive measures, making timely diagnoses, and providing prompt treatment of CVD risk factors.

Health Education

In order to enable people to make knowledgeable decisions about their health and adopt healthier habits, health education is essential. Targeted health education interventions aim to increase the awareness of cardiovascular risk factors, promote lifestyle modifications, and facilitate adherence to preventive measures [14-16]. By empowering people with the knowledge and abilities needed to manage their cardiovascular health, health education programs can improve long-term health outcomes and reduce the risk of developing cardiovascular disease (CVD). Numerous observational and randomized controlled trials have demonstrated the effectiveness of health education programs in lowering cardiovascular risk factors and enhancing cardiovascular outcomes in a range of demographics. Such programs often incorporate a multidisciplinary approach, including components such as dietary counseling, physical activity promotion, stress management techniques, and smoking cessation support. By addressing modifiable risk factors and promoting behavioral changes, health education interventions have demonstrated significant reductions in blood pressure, cholesterol levels, body weight, and overall cardiovascular risk [17,18].

Yoga Therapy

Yoga therapy has emerged as a promising complementary intervention for managing cardiovascular disease risk factors and promoting overall heart health. This holistic approach includes various relaxation techniques such as meditation, mindfulness, and deep breathing, which can help lower stress levels, which are significant risk factors for cardiovascular disease (CVD). Additionally, yoga's physical postures (asanas) contribute to weight management, improved circulation, and improved cardiovascular fitness, all of which are crucial factors for reducing the risk of CVD [19-22]. A review of the available literature found numerous substantial drawbacks and gaps, including methodological inconsistencies, limited sample numbers, and the lack of long-term follow-up assessments, despite the encouraging outcomes seen in several studies. The robustness and generalizability of the results are restricted by these limitations. Furthermore, the dearth of studies focusing on police populations highlights the necessity for more research on customized therapies that address the particular demands and difficulties experienced by law enforcement officers. Yoga encourages a holistic approach to health, often including recommendations for dietary improvements, smoking cessation, and other lifestyle changes beneficial to heart health. Mental health is intimately tied to cardiovascular health, and yoga can enhance emotional well-being, alleviate the symptoms of depression and anxiety, and promote overall mood improvement. All of these factors work together to benefit the heart. A review of the available literature found numerous substantial drawbacks and gaps, including methodological inconsistencies, limited sample numbers, and the lack of long-term follow-up assessments, despite the encouraging outcomes seen in several studies. The articles included in the study have been summarized based on author name, year of publication, body parameters measured, and key findings of the studies in Table 1.

**Table 1:** Summary of the articles included in the review (n=8)

Authors	Year	Title	Key findings	Limitations
Sharma et al. [23]	2015	Effect of yoga and health education on stress and cardiovascular risk factors among police personnel	Yoga and health education significantly reduced stress and improved cardiovascular risk factors	Small sample size, short duration of intervention
Singh et al. [24]	2016	Cardiovascular benefits of yoga: a review of studies in Indian police personnel	Yoga was associated with improved cardiovascular health markers, including reduced blood pressure and cholesterol levels	Lack of control groups
Patel et al. [25]	2017	Impact of yoga on cardiovascular health in Indian police personnel	Significant improvements in cardiovascular health parameters, including lower incidence of hypertension	High dropout rate, varied adherence to yoga practice
Kumar et al. [26]	2018	Health education and yoga interventions for cardiovascular health evidence from police personnel	Combined interventions led to a notable reduction in cardiovascular risk factors among police personnel	Short follow-up period, self-reported data
Joshi et al. [27]	2019	Yoga and cardiovascular health in police personnel	Yoga practice resulted in better cardiovascular outcomes compared to standard health education alone	Limited generalizability, regional differences
Reddy et al. [28]	2020	Effectiveness of yoga-based stress management on cardiovascular health among police personnel	Yoga-based stress management effectively reduced stress and improved cardiovascular health indicators	Small sample size, lack of long-term follow-up
Gupta et al. [29]	2021	Integration of yoga and health education for cardiovascular health in police personnel	Yoga combined with health education showed improvements in cardiovascular health and overall well-being	Variability in intervention protocols
Bhatia et al [30]	2022	Long term effects of yoga and health education on cardiovascular health among police personnel in India	Long-term yoga practice led to sustained improvements in cardiovascular health and reduced risk factors	Potential biases due to selection

The robustness and generalizability of the results are restricted by these limitations. Furthermore, the dearth of studies focusing on police populations highlights the necessity for more research on customized therapies that address the particular demands and difficulties experienced by law enforcement officers. Nevertheless, it is crucial to reiterate that yoga therapy should always be integrated with conventional medical care and supervised by qualified healthcare professionals. Patients with pre-existing cardiovascular conditions are strongly advised to consult their healthcare providers before initiating any new exercise regimen, including yoga. Moreover, not all forms of yoga may be suitable for everyone; thus, it is essential to identify a style and intensity level that aligns with individual needs and abilities. The results of body parameters that have been increased or decreased with the reason behind the changes have been listed in Table 2.

**Table 2:** Impact of yoga therapy and health education among the body parameters LDL - low-density lipoprotein; HDL - high-density lipoprotein

Authors	Year	Parameters checked	Parameters increased	Parameters decreased	Reason for changes among body parameters
Sharma et al.	2015	Blood pressure, cholesterol levels, heart rate variability	Heart rate variability	Blood pressure, cholesterol levels	Regular yoga practice improved autonomic function.
Singh et al.	2016	Blood pressure, cholesterol levels, LDL, Triglycerides	N/A	Blood pressure, cholesterol levels, LDL, Triglycerides	Yoga's emphasis on relaxation and stress reduction led to lower blood pressure and lipid levels.
Patel et al.	2017	Blood pressure, fasting blood glucose, LDL cholesterol, HDL cholesterol	HDL cholesterol	Blood pressure, fasting blood glucose, LDL cholesterol	Regular yoga improved lipid profiles by increasing HDL cholesterol and decreasing LDL and blood glucose due to enhanced metabolic function.
Kumar et al.	2018	Body mass index (BMI), waist-to-hip ratio, blood pressure, physical fitness level	Physical fitness level	Body mass index (BMI), waist-to-hip ratio, blood pressure	Combined interventions of yoga and health education promoted physical activity and dietary changes, leading to improved BMI and cardiovascular health.
Joshi et al.	2019	Blood pressure, cholesterol levels, exercise tolerance	Exercise tolerance	Blood pressure, cholesterol levels	Yoga increased exercise tolerance by improving cardiovascular efficiency and reducing stress, leading to decreased blood pressure and cholesterol.
Reddy et al.	2020	Resting heart rate, blood pressure, stress resilience	Stress resilience	Resting heart rate, blood pressure	Yoga-based stress management enhanced stress resilience, leading to lower resting heart rate and blood pressure.
Gupta et al.	2021	Blood pressure, fasting glucose, waist circumference, quality of life	Quality of life	Blood pressure, fasting glucose, waist circumference	Integrating yoga with health education improved overall well-being, reducing stress-related parameters like blood pressure and waist circumference
Bhati et al.	2022	Blood pressure, cholesterol levels, long-term health compliance	Long-term health compliance	Blood pressure, cholesterol levels	Sustained yoga practice fostered long-term adherence to healthy behaviors, resulting in ongoing reductions in cardiovascular risk factors.

Research conducted in other countries has shown promising outcomes for yoga and health education in enhancing cardiovascular health among various occupational groups, but the specific impact on police personnel in South India remains understudied. For instance, studies in Western nations, such as the United States and the United Kingdom, have demonstrated that regular yoga can significantly decrease stress and improve heart health among law enforcement officers. Similarly, health education initiatives in countries like Canada have been effective in promoting cardiovascular wellness. However, the unique cultural, occupational, and environmental factors in South India require a tailored approach. This scoping review aims to address this gap by examining the effectiveness of these interventions in the South Indian context, thereby contributing to a more comprehensive understanding of global best practices and their applicability in diverse settings. This study aimed to address these gaps by evaluating the feasibility, acceptability, and sustainability of implementing health education and yoga therapy programs tailored to the unique requirements and preferences of police officers in South India. Early detection and management of hypertension, dyslipidemia, and diabetes are vital for reducing the risk of cardiovascular disease and preventing complications. This study sought to assess the effects of a comprehensive intervention on major cardiovascular risk factors among participating police officers, including blood pressure, lipid profile, body mass index, and psychological well-being. The intervention consisted of yoga training and health education sessions. In South India, where CVDs are prevalent and yoga is deeply ingrained in traditional culture, it is beneficial to explore the potential of health education and yoga interventions to reduce the risk of CVDs among police personnel. This approach could provide a promising opportunity to address this pressing public health challenge effectively. 

Significance of the study

Police officers constitute a critical workforce subject to high levels of stress and demanding job conditions, which increases their risk of cardiovascular diseases. Therefore, it is essential to investigate the effects of interventions, such as yoga and health education, to develop targeted strategies for improving cardiovascular health. This study not only addresses a significant public health concern but also adds to the necessary evidence base for implementing effective health promotion programs. The results of this investigation can inform policy decisions and ultimately lead to better health outcomes for police officers in South India.

Limitations

The limitations of the studies included in this review must be acknowledged, primarily because they were conducted primarily in India, which restricts the generalizability of the findings to other cultural contexts. The practices of yoga and health education programs vary widely, resulting in small sample sizes and the absence of randomized controlled designs in some studies, thereby weakening the quality of evidence. Most studies had short follow-up periods, limiting the ability to evaluate long-term effects. Furthermore, reliance on self-reported data, potential publication bias, and the lack of standardized outcome measures impose constraints on the conclusions that can be drawn. Although this review focuses on cardiovascular health, it is important to recognize the potential positive impact of yoga and health education on mental health, injury prevention, and job performance among police personnel. Future research should address these limitations to provide more robust and comprehensive insights into the benefits of these interventions for police personnel.

Implications for future research

The significance of conducting comprehensive and long-term research on the consequences of yoga and health education on cardiovascular health among police personnel is highlighted in this scoping review. Future studies should prioritize the use of longitudinal designs to evaluate sustained benefits, incorporate diverse demographic groups within the police force, and conduct comparative analyses with other physical activities or stress management interventions. Investigating the biological and psychological mechanisms that support yoga's impact on cardiovascular health, as well as integrating holistic practices, such as diet and mindfulness, could provide deeper insights. Furthermore, exploring the use of technology, such as wearable devices, to promote adherence and monitor progress, along with cost-effectiveness analyses, would be a valuable area of inquiry. 

## Conclusions

By comprehensively analyzing the existing literature, this scoping review study uncovered essential discoveries that emphasized both the need for improvement and gaps in the current understanding. A review of the available literature found numerous substantial drawbacks and gaps, including methodological inconsistencies, limited sample numbers, and the lack of long-term follow-up assessments, despite the encouraging outcomes observed in several studies. These limitations restrict the robustness and generalizability of the results. Furthermore, the dearth of studies focusing on police populations highlights the necessity for more research on customized therapies that address particular demands and difficulties experienced by law enforcement officers. In conclusion, this scoping review sheds light on the potential of yoga and health education interventions in promoting cardiovascular health among police personnel. Although the current evidence suggests positive outcomes, future research should focus on enhancing methodological rigor, increasing sample sizes, and conducting longitudinal evaluations to determine the long-term effectiveness and scalability of these interventions within the law enforcement community. Addressing these research gaps will not only advance scientific understanding but also inform the development of targeted interventions aimed at safeguarding the cardiovascular health of those who serve and protect our communities.
